# Polyhydroxybutyrate / carbonized waste rubber biocomposite films

**DOI:** 10.1038/s41598-026-45256-z

**Published:** 2026-03-23

**Authors:** Ferhat Şen, Mustafa Zor, Zeki Candan, Deniz Aydemir, Davood Peyrow Hedayati, Saskia Roßberg, Andrea Berlich, Robert Böhm

**Affiliations:** 1 Department of Nanotechnology Engineering, Zonguldak Bülent Ecevit University, Zonguldak, Türkiye; 2 Biomaterials and Nanotechnology Research Group & BioNanoTeam, İstanbul, Türkiye; 3 Department of Forest Industrial Engineering, İstanbul University-Cerrahpaşa, İstanbul, Türkiye; 4 Department of Forest Industrial Engineering, Bartın University, Bartın, Türkiye; 5 Faculty of Engineering, Leipzig University of Applied Sciences (HTWK Leipzig), Leipzig, Germany; 6 Centre of Mathematics and Natural Science, Leipzig University of Applied Sciences (HTWK Leipzig), Leipzig, Germany

**Keywords:** Polyhydroxybutyrate, Carbonized Waste Rubber, Biocomposites, Sustainability, Chemistry, Energy science and technology, Engineering, Materials science

## Abstract

The utilization of waste tires in combination with biodegradable polymers offers an innovative approach to sustainable material production. This strategy provides significant advantages in both environmental sustainability and functional performance. In this study, it was aimed to manufacture and characterize novel biocomposites based on the biopolymer polyhydroxybutyrate (PHB) reinforced with carbonized materials obtained by pyrolysis of waste rubbers. PHB biocomposite films containing 0.5%, 1% and 2% carbonized waste rubber (CWR) by weight were prepared by solvent casting method. The thermal properties of those new biocomposite films were examined with TGA and DSC techniques, and their structural and morphological properties were examined with IR microscopy and SEM techniques. Additionally, the electrical conductivity of biocomposites was determined. All the results obtained showed that CWR addition increased the ash yield of biocomposite films, and the highest electrical conductivity was achieved with 1% CWR addition. These findings suggest that incorporating carbonized waste rubber into PHB enhances the material’s thermal stability and electrical conductivity, making it a promising candidate for sustainable and high-performance biocomposite applications.

## Introduction

 In recent years, the use of petrochemical-derived plastics in many application areas has increased, which pose significant threats to human and environmental health. Although the use of traditional petrochemical-derived plastic products has improved the quality of daily life, it has also led to the accumulation of waste that is constantly increasing. This shows the need of material alternatives like biodegradable plastics^1^.

Poly (3-hydroxybutyrate) (PHB), a biodegradable polymer, is known for its ease of processing, its high biocompatibility, and non-toxic degradation products, and is produced by many bacteria as a natural polymer for energy storage. However, it is known that PHB contains some difficulties such as its brittle fracture behaviour resulting from its apparent crystallinity, high production cost, unclear mechanical strengths limit its usability in technical applications^2–5^. Although PHB exhibits a relatively high melting temperature (approximately 180 °C), its thermal processing is often limited by its narrow processing window, since thermal degradation can occur close to its melting temperature. PHB typically starts to thermally decompose at temperatures around 280–300 °C, which is a critical parameter influencing its processing stability and end-use performance. Therefore, improving the thermal processability of PHB without significantly reducing its thermal stability is an important research focus.

While PHB is a good candidate material for green applications such as packaging, it has significant flaws that prevent it from being widely used in the packaging industry. Due to the phenomenon of crystallization and physiological aging at room temperature, PHB has a relatively high instability, which increases with time. PHB also has a small processing range, making it difficult to handle in some typical packaging applications such as heat treatment^6^. Another important obstacle to a wider application area in the packaging industry is its high cost. In this context, the inclusion of a long-lasting, low-cost, or hard fillers can help mitigate the crude price increase by (a) minimizing the overall packaging cost and/or (b) reducing the required thicknesses in standard packaging. PHB, a new generation biopolymer, can be used as an engineering material in the future. Scientific studies have been conducted to improve the mechanical properties of this biopolymer, reduce its brittleness, and improve its thermal instability with several modification techniques^7–9^. Recently, studies have been carried out with PHB biocomposites to increase their thermal stability^10,11^. PHB polymer composites, cellulose nanocrystals^10^, agave fibers^12^, chitosan^13^, graphene^11^, American rhea eggshell^14^ were used and characterized. All results indicate that PHB biocomposites can be used for future applications in industry.

Safe processing and recycling of waste tires have always been the focus and challenge of the global rubber industry. Pyrolysis not only solves the problem of environmental pollution, but also can completely purify waste tires and recover valuable pyrolysis products. Tires made from rubber and various rubber products are widely used around the world, resulting in large amounts of difficult-to-decompose waste rubber^15–22^. In the study conducted by Kocatürk et al.^23^, carbonized waste rubber obtained by pyrolysis of tire waste was reinforced with nanocellulose at the rates of 0.10%, 0.25%, 0.5% and 1% by weight. The prepared nanocellulose-based nanocomposites were examined by X-ray diffraction (XRD), morphological properties by scanning electron microscope (SEM), thermal properties by thermogravimetric analysis (TGA), differential scanning calorimetry (DSC) and dynamic mechanical thermal (DMTA) methods. There are studies on nanocellulose and its derivatives to strengthen carbonized waste rubber material, but the usability of PHB carbonized waste rubber material as reinforcement to increase thermal stability has not been tested.

In our study, we have produced and characterized polyhydroxybutyrate / carbonized waste rubber biocomposite films formed by combining polyhydroxybutyrate and carbonized waste rubber (CWR) obtained by carbonization of recycled tire waste. Thermal gravimetric analysis (TGA), differential scanning calorimetry (DSC), structural characterization (IR microscopy), electrical conductivity and scanning electron microscope (SEM) images of biocomposite films were examined to characterize the properties of the novel biocomposite.

## Experimental

### Materials

Polyhydroxybutyrate pellets (PHB, Mw: 190 kDa, BRS Bulk Bio-Pellets) were supplied from Bulk Reef Supply, Golden Valley, MN, EUA. Carbonized waste rubber (CWR) material by pyrolysis method under high pressure was produced and supplied by ZBB GmbH (Germany). Chloroform (99%) was purchased from Sigma Aldrich.

### Preparation of the biocomposite films

PHB (Polyhydroxybutyrate), CWR and 70 mL of chloroform in the amounts specified in Table 1 were taken into a beaker and stirred for 2 h at the boiling point of chloroform. The mixture was kept in an ultrasonic bath for 30 min to ensure homogeneous distribution of CWR in different proportions by mass. The homogeneous mixture was poured into a petri dish and composite materials were obtained by removing chloroform at room temperature under a fume hood.

**Table 1:** Formulation of biocomposite materials.

Sample	Polyhydroxybutyrate (PHB) (g)	Carbonized Waste Rubber (CWR) (g)	Carbonized Waste Rubber (CWR) (%)
F0	5	-	-
F1	5	0.025	0.5
F2	5	0.05	1
F3	5	0.1	2

### Characterization and measurements

TGA (Hitachi STA 7300, Tokyo, Japan) was carried out under nitrogen atmosphere with a heating rate of 25 °C per minute between 40 and 750 °C.

Shimadzu DSC-60 differential scanning calorimeter device was used for analysis. Approximately 15 mg of the sample was placed in an aluminum pan. DSC analysis was performed from 30 °C to 600 °C with heating at 10 °C per minute. During the analysis, 50 mL of nitrogen gas was given to the environment per minute.

Fluke 45 Dual Display Multimeter was used to determine the electrical conductivity of biocomposite films. The electrical resistance of 6 cm long samples was measured in ohm at room temperature.The morphology of the biocomposite films was observed by scanning electron microscopy (Prisma E, Thermo Fisher Scientific Inc., Waltham, USA). Samples were immersed in liquid nitrogen, then cut to obtain the cross section and finally coated with gold using a sputter coater. The electrical conductivity of biocomposite films was calculated using the measured electrical resistance and sample geometry according to the following equation:

where σ is the electrical conductivity (S/m), L is the distance between electrodes (m), R is the measured electrical resistance (Ω), and A is the cross-sectional area of the film calculated as the product of film thickness and width. The electrode distance was kept constant at 60 mm for all samples.

FT-IR was used to determine the changes in the chemical structure of the different biopolymers. Each spectrum was recorded in the range of 650–4000 cm^− 1^ with a resolution of 2 cm^− 1^. Perkin Elmer Spectrum One model FTIR spectrophotometer was used for this analysis.

The morphology of the biocomposite films was examined by SEM. The samples were cut and plated with gold. The morphology of the cut surfaces of the samples was examined.

## Results and discussion

### Thermal properties of biocomposite films

**Fig. 1:**
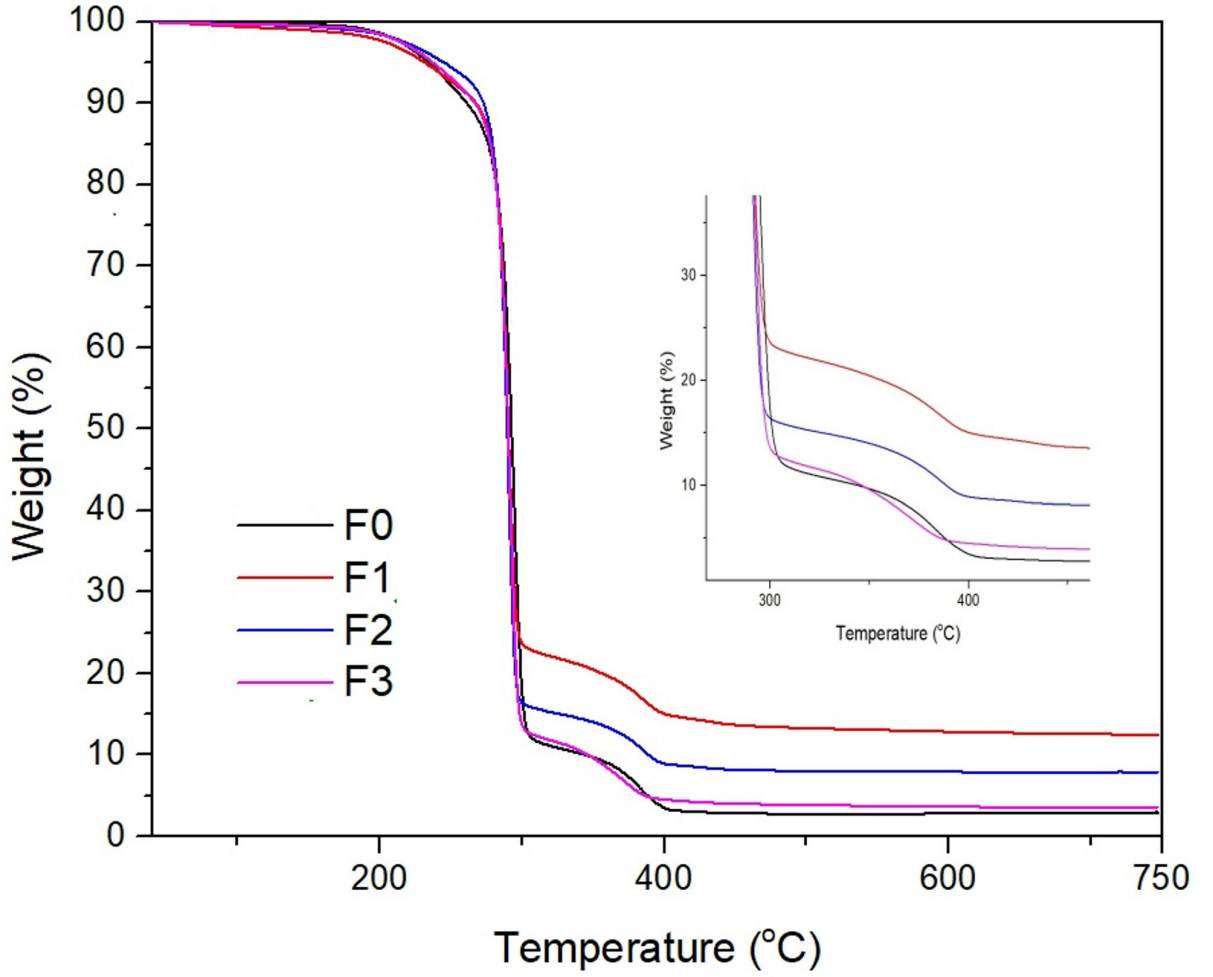
TGA of biocomposite films.

Thermograms of the biocomposites are shown in Fig. 1, and the results obtained from the thermograms are shown in Table 2. The results obtained show that the TGA-DTG curves of the biocomposites exhibit three different degradation stages. The initial weight loss observed between 180 and 260 °C (10% of its mass) may be attributed to early thermal degradation of PHB, including random chain scission and ester bond cleavage reactions, together with the possible evaporation of residual or bound moisture within the polymer matrix^24^. The second degradation is due to thermal degradation of the polymer main chains of the biocomposites. The main decomposition occurred at approximately 290 °C, and at this stage the samples lost 80–90% of their mass. It appears that the CWR contribution does not have a significant effect on the main decomposition temperature. The third degradation stage is caused by CWR used as a reinforcement material. This decomposition occurs between 300 and 400 °C, and at this stage the samples lost approximately 20% of their mass. Additionally, when the remaining ash amounts of the samples at 750 °C are examined, it is found that the ash amount of the FO sample is 2.90% and the F3 sample containing 2% CWR is 12.40%. These results show that the ash yield clearly increases with the increase in the amount of CWR in the biocomposite.

**Table 2:** Thermal properties of biocomposite films.

Sample	T_10%_ (ºC)	Max. weight loss (ºC)	Char yield (%)
F0	261	294	2.90
F1	266	292	3.51
F2	272	290	7.88
F3	267	290	12.40

**Fig. 2:**
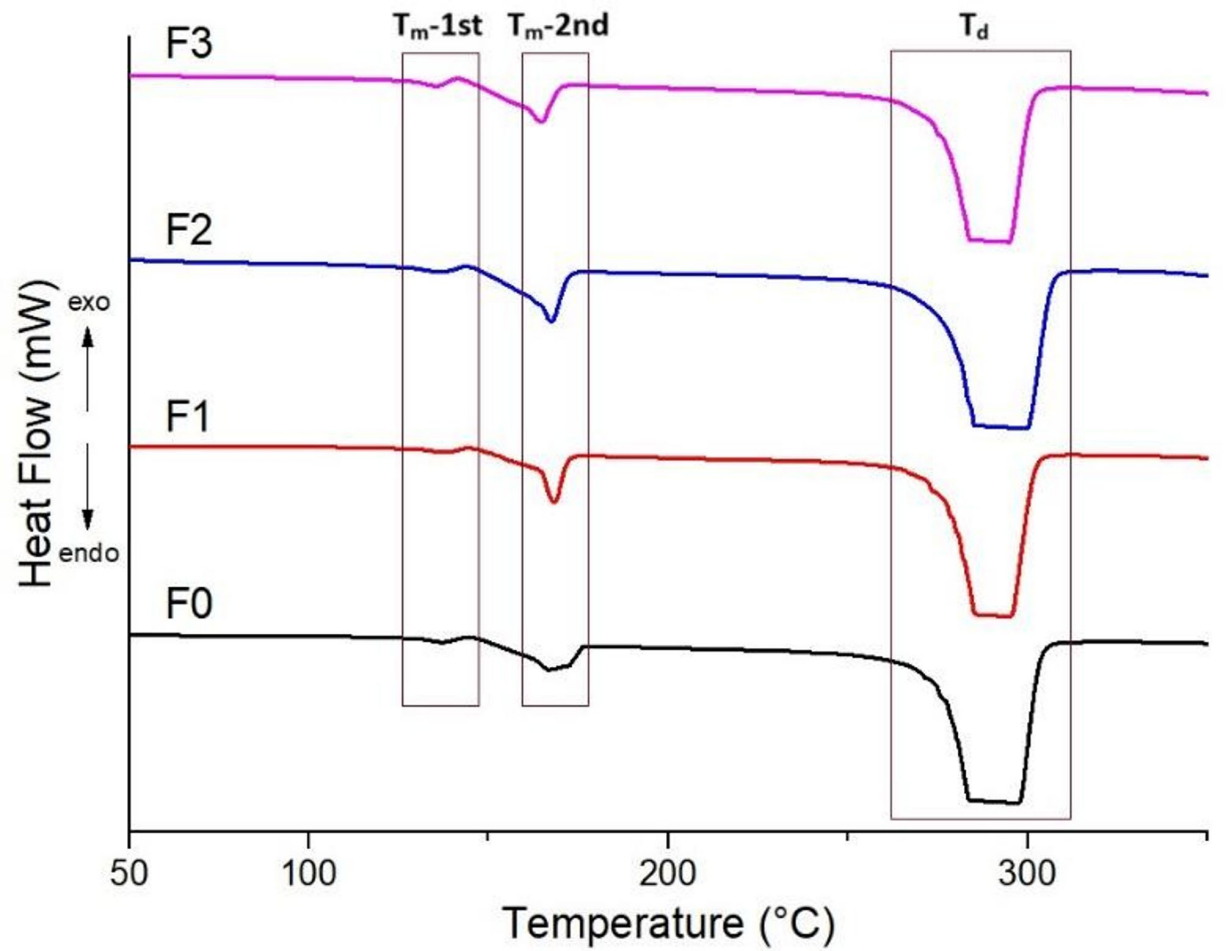
DSC results of biocomposite films.

The DSC results of biocomposite films are summarized in Fig. 2, and the data is summarized in Table 3. According to the DSC results, two different endothermic peaks were detected. These peaks are peaks belonging to melting points. The melting points of the samples were determined between 135 and 168 °C. Additionally, a broad range of endothermic peaks were detected at rising temperatures. These peaks indicate the degradation temperatures and are between 283 and 300 °C. The melting temperature values ​​of the PHB-based samples did not show significant changes compared to pure PHB; this indicates that the crystalline structure of the polymer is largely preserved. The crystallinity of pure PHB (F0) was determined to be 8.19%, while a slight increase was observed in composites containing carbonized waste rubber, reaching 9.00% for F2. The increase in crystallinity can be attributed to the nucleating effect of carbonized waste rubber particles, which can promote crystal formation^25^. However, the overall changes remained limited, indicating that the crystalline structure of PHB was largely preserved.

**Table 3:** DSC results of biocomposite films.

Samples	T_m_ (ºC)		T_d_ (ºC)	X_c_ (%)
	1st	2nd		
F0	137	166	283–298	8.19
F1	137	168	285–295	8.55
F2	136	167	285–300	9.00
F3	135	165	283–295	8.86

### Electrical properties of biocomposite films

The electrical conductivity of the new biocomposite materials is examined, and shown in Table 4. It was found that the electrical conductivity increases with increasing CWR content to the composites. However, it was determined that more than 1% weight CWR caused a decrease in the electrical conductivity. The decrease in electrical conductivity observed at CWR contents above 1% is likely due to the agglomeration of carbonized particles within the PHB matrix. At higher loadings, carbon particles tend to cluster, disrupting the conductive network continuity, and leading to a lower overall conductivity despite the higher filler content. Similar behavior has been reported in other polymer/carbon composites^26^. The electrical conductivity values ​obtained for PHB-based composites range from 2.60 × 10^− 4^ to 6.25 × 10^− 4^ S/m. According to commonly accepted conductivity classifications, materials with conductivity values ​​between 10^− 12^ and 10^− 6^ S/m are considered antistatic, while materials with conductivity values ​​between 10^− 6^ and 10^4^ S/m are classified as semiconductors. According to this classification, the developed composites clearly fall into the semiconductor region^27^.

**Table 4:** Electrical conductivity measurements of biocomposite films considering sample geometry at room temperature.

Sample	*R* calculated (Ω)	Length (mm)	Thickness (mm)	Width (mm)	Electrical conductivity (S/m)
F0	70 × 10^6^	60	0,33	10	2.60 × 10^− 4^
F1	45 × 10^6^	60	0,30	10	4.44 × 10^− 4^
F2	30 × 10^6^	60	0,32	10	6.25 × 10^− 4^
F3	40 × 10^6^	60	0,32	10	4.69 × 10^− 4^

### Structural characterization of biocomposite films

**Fig. 3:**
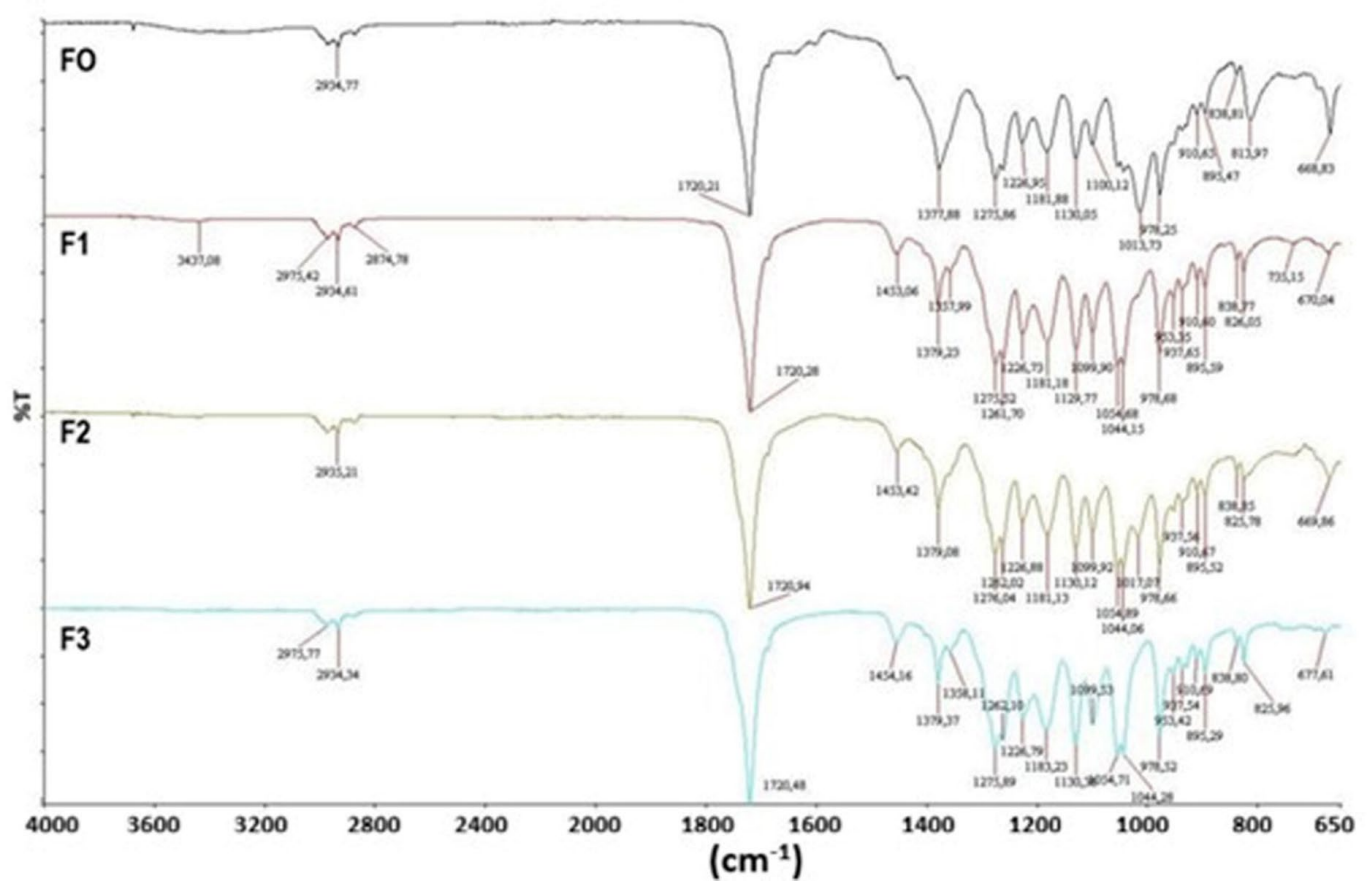
FT-IR spectra of the biocomposite films.

FT-IR spectra of the biocomposite films are shown in Fig. 3. Slight changes have been noticed in the FTIR spectra of PHB/CWR biocomposites, particularly in the bands of –C–H– and C = O stretching vibrations. A minor shift in absorption peaks observed at 1379 cm⁻¹ and 1720 cm⁻¹ may be due to weak intermolecular interactions, possibly due to hydrogen bonding and changes in the molecular environment of the PHB chains due to the incorporation of CWR. Changes in the intensity of characteristic absorption bands of PHB, such as C = O stretching and –C–O–C– vibrations at 1190 cm⁻¹, 1226 cm⁻¹, and 1276 cm⁻¹, suggest that there are slight changes in the level of crystallinity of the polymer matrix. Absorption bands observed at 2935 cm⁻¹ and 2975 cm⁻¹ correspond to symmetric and asymmetric stretching vibrations of methyl and methylene groups, which are characteristic of the PHB structure^28^. FTIR spectra of composite film samples F1, F2, and F3 show significant similarity to that of neat PHB, suggesting that incorporation of CWR does not significantly affect the chemical structure of the polymer matrix.

### Morphology of biocomposite films

As shown in Fig. 8, pores were observed on the SEM images of the biocomposites. These pores are related to the high viscosity and durability of the PHB matrix. It was determined that the CWR reinforcement was homogeneously distributed within the PHB matrix, and that the matrix and the reinforcement were compatible with each other. The solvent casting technique used in this study can affect the structural and morphological properties of PHB-based composites. Solvent evaporation during film formation can lead to the formation of porous structures and affect the polymer chain arrangement and crystallinity. These effects are also supported by SEM observations showing porous morphology in the produced films. Compared to melt processing methods such as extrusion or compression molding, solvent casting typically produces films with higher porosity and different crystallization behavior due to slower solvent evaporation and lower shear forces^29^. Therefore, the solvent casting approach was preferred in this study to maintain polymer stability and ensure homogeneous distribution of carbonized waste rubber.

**Fig. 4:**
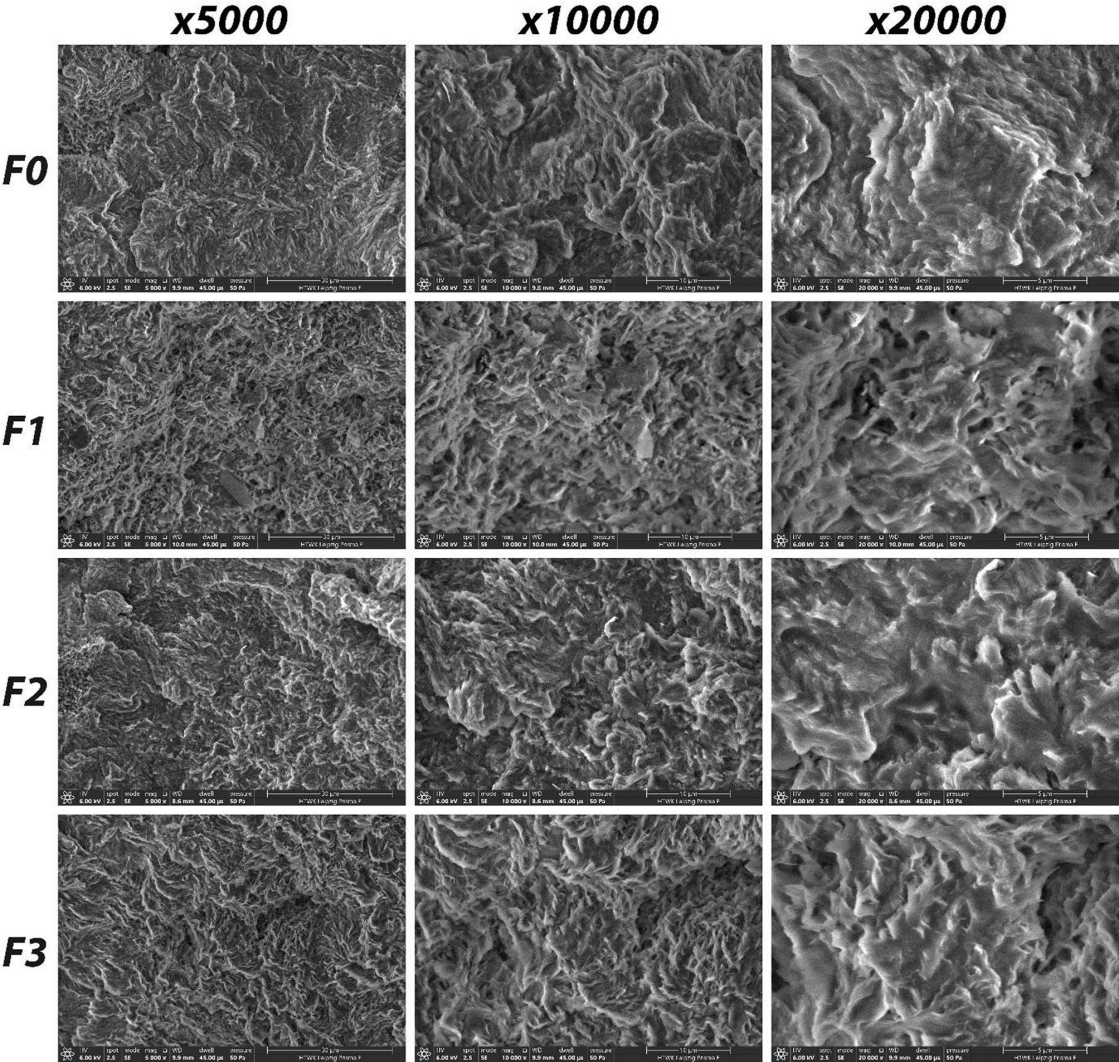
SEM images of biocomposite films.

## Conclusions

In this study, PHB biocomposite films containing different ratios of carbonized waste rubber were successfully produced and characterized. TGA results showed that the biocomposite films degraded in three stages. It was determined that the CWR additive clearly increased the ash yield. According to DSC results, the melting points of the samples were determined to be between 135 and 168 °C and the decomposition temperatures were between 283 and 300 °C. It was observed that the CWR contribution had no effect on these thermal transitions. It has been observed that CWR additives of up to 1% increases the electrical conductivity of the biocomposites. IR microscopy results showed that all samples were homogeneous, and all samples had similar spectra. SEM images showed that the biocomposites had a porous structure and the CWR reinforcement was distributed homogeneously in the PHB. As a result, instead of disposing of waste tires, they can be carbonized and converted into industrial products, and by continuing studies in this field, a contribution can be made to the sustainable economy.

## Data Availability

The datasets used and/or analysed during the current study available from the corresponding author on reasonable request.
